# Hypoglycemic Toxins and Enteroviruses as Causes of Outbreaks of Acute Encephalitis-Like Syndrome in Children, Bac Giang Province, Northern Vietnam

**DOI:** 10.3201/eid2408.171004

**Published:** 2018-08

**Authors:** Nga Thi Phan, Meriadeg Ar Gouilh, Juliette Paireau, Loan Phuong, Justine Cheval, Nghia Duy Ngu, Charles Hébert, Tuan Hai Nguyen, Olivier Lortholary, Laura Tondeur, Jean-Claude Manuguerra, Robert Barouki, Johannes Sander, Nils Janzen, Hien Tran Nguyen, Paul T. Brey, Arnaud Fontanet, Marc Eloit

**Affiliations:** National Institute of Hygiene and Epidemiology, Hanoi, Vietnam (N.T. Phan, L.P. Do, N.D. Ngu, T.H. Nguyen, H.T. Nguyen);; Institut Pasteur, Paris, France (M. Ar Gouilh, J. Paireau, L. Tondeur, J.-C. Manuguerra, A. Fontanet, M. Eloit);; Centre National de la Recherche Scientifique, Paris (J. Paireau);; Pathoquest, Paris (J. Cheval, C. Hébert, M. Eloit);; Hôpital Necker-Enfants Malades, Paris (O. Lortholary, R. Barouki);; Université Paris Descartes, Paris (O. Lortholary, R. Barouki);; **Institut National de la Santé et de la Recherche Médicale,** Paris (R. Barouki);; Screening Laboratory Hannover, Hannover, Germany (J. Sander, N. Janzen);; Hannover Medical School, Hannover (N. Janzen);; Institut Pasteur du Laos, Vientiane, Laos (P.T. Brey); Conservatoire National des Arts et Métiers, Paris (A. Fontanet)

**Keywords:** hypoglycemic toxins, enteroviruses, viruses, meningitis/encephalitis, encephalitis, acute encephalitis syndrome, acute encephalitis-like syndrome, hypoglycemia, outbreaks, children, hypoglycins, litchi, litchi cultivation, methylenecyclopropylglycine, Bac Giang Province, Vietnam

## Abstract

We investigated the cause of seasonal outbreaks of pediatric acute encephalitis-like syndrome associated with litchi harvests (May–July) in northern Vietnam since 2008. Nineteen cerebrospinal fluid samples were positive for human enterovirus B, and 8 blood samples were positive for hypoglycemic toxins present in litchi fruits. Patients who were positive for hypoglycemic toxins had shorter median times between disease onset and admission, more reports of seizures, more reports of hypoglycemia (glucose level <3 mmol/L), lower median numbers of leukocytes in cerebrospinal fluid, and higher median serum levels of alanine aminotransferase and aspartate transaminase than did patients who were positive for enteroviruses. We suggest that children with rapidly progressing acute encephalitis-like syndrome at the time of the litchi harvest have intoxication caused by hypoglycemic toxins, rather than viral encephalitis, as previously suspected. These children should be urgently treated for life-threatening hypoglycemia.

Acute encephalitis syndrome is clinically characterized by fever, seizures, and altered mental status. This syndrome is a major public health concern in Asia; annually, >133,000 children are hospitalized with this pathology (*1*,*2*). Historically, the main etiology of acute encephalitis syndrome in Asia has been Japanese encephalitis, a vectorborne disease caused by a flavivirus (Japanese encephalitis virus)*,* which causes >25% of cases of this syndrome in Asia. Many other viruses have been shown to cause acute encephalitis syndrome in Asia, such as enteroviruses (e.g., poliovirus, echovirus 9, enterovirus 71), and herpes simplex, measles, varicella zoster, rabies, dengue, Chandipura, and Nipah viruses.

However, for most cases of acute encephalitis syndrome, the specific etiology is unknown (*3*–*5*). Since introduction of Japanese encephalitis vaccine in the Expanded Program on Immunization in Asia (South Korea, China, Bangladesh, and Nepal) in 1997, a major shift has occurred; cases of Japanese encephalitis–attributable acute encephalitis syndrome have decreased, and cases of acute encephalitis syndrome not attributed to Japanese encephalitis have increased in countries with large vaccination coverage (*6*–*10*).

Such a shift has been observed in Vietnam, where the prevalence of Japanese encephalitis for hospitalized patients decreased from 50% in 1996 to 10% in 2009 (T.P. Nga, unpub. data). According to the Ministry of Health, 67% of the 1,800–2,300 cases of acute encephalitis syndrome reported each year occur in northern Vietnam, most often in Bac Giang Province (http://moh.gov.vn/Pages/Search.aspx?Key=japanese encephalitis in Vietnam). There has been a clear seasonal pattern of acute encephalitis-like syndrome in this province since 1999, with peaks in summer, particularly in young children (*11*). The symptomatology described by parents as rapid development of fever, headache, and nocturnal seizures explains the local name given to the disease (Ac Mong, meaning nightmare). Local populations had previously suggested a link with litchi cultivation because of observed synchronicity between outbreaks of acute encephalitis-like syndrome and litchi harvests.

A previous investigation of outbreaks of acute encephalitis-like syndrome in Bac Giang Province found that litchi cultivation appeared to be associated with acute encephalitis-like syndrome, but the link at the individual level remained unclear (*11*). Until 2007, results of all virologic investigations on patient samples remained inconclusive. We therefore performed next-generation sequencing (NGS) on cerebrospinal fluid (CSF) samples obtained since 2008 to identify unknown or unforeseen viruses. In addition, because hypoglycin A (HGA) and methylenecyclopropylglycine (MCPG) are suspected to be probable causes of similar outbreaks of acute encephalitis syndrome during litchi harvests in India and Bangladesh (*12*–*16*), we also tested serum samples for these toxins. These toxins are present in seeds and aril (flesh) of litchis (*17*,*18*) and are known to induce hypoglycemia in animal models (*19*).

## Materials and Methods

### Study Area and Outbreak Characteristics

Detailed characteristics for this study have been previously reported (*11*). In brief, Bac Giang Province is a rural province that has 1.6 million inhabitants and is located in northern Vietnam. The only hospital is located in Bac Giang City, the capital of the province. Outbreaks of acute encephalitis-like syndrome in Bac Giang Province are unusual and characterized by their specific location, strict seasonality, restricted age group, rapid progression to coma, and a higher case-fatality rate than that for Japanese encephalitis (*11*).

### Study Design and Data Collection

We used surveillance data for case-patients with acute encephalitis-like syndrome admitted to the Bac Giang Provincial Hospital during 2008–2011. The case definition used for this syndrome was fever (temperature >37°5C reported by parents or at hospital admission), altered mental status or seizures, and no bacterial meningitis. We collected data from the Bac Giang Preventive Medicine Centre and the National Institute of Hygiene and Epidemiology (Hanoi, Vietnam). Patients <15 years of age, those who had onset of acute encephalitis-like syndrome during May 1–August 31, and those who had negative results for Japanese encephalitis virus IgM in CSF or were immunized against Japanese encephalitis virus were included in the study.

### CSF and Blood Samples

For each seasonal outbreak that occurred during 2008–2011, blood samples were obtained from patients with acute encephalitis syndrome at admission to Bac Giang Provincial Hospital for standard biochemical and hematologic analysis. CSF samples from patients were also collected by physicians. Samples were cryopreserved in liquid nitrogen for transportation and stored at −80°C. Because most patients were young children, only small volumes (20 μL–300 μL) of CSF were available for most samples. Because of age of patients and local cultural practices, no brain biopsies or necropsies were performed for children who died of acute encephalitis-like syndrome.

### Virologic Analyses

We conducted virus isolation in RD, Vero E6, or C6/36 monolayer cells and PCR for known viruses (Technical Appendix). In addition, we also performed random NGS for 16 selected CSF samples that matched the case definition and were collected during 2008. We performed specific PCRs for contigs identified by NGS to confirm results, which also enabled comprehensive phylogenetic analysis by using other methods (Technical Appendix). Primers (Technical Appendix Table 1) were used to test CSF samples obtained from patients who had available clinical data (3 in 2008, 3 in 2009, 14 in 2010, and 21 in 2011).

### Toxicologic Analysis

We tested 20 blood samples obtained during 2010–2011 (4 in 2010 and 16 in 2011, the only ones available from patients who had clinical data at the time when the hypoglycemic toxins hypothesis was proposed) for HGA and its metabolites and metabolites of MCPG by using a modified analytical method that has been reported (*20*,*21*). This method is based on ultra–high-performance liquid chromatography/tandem mass spectrometry.

Because these toxins can cause hypoglycemia by blocking the fatty acid β-oxidation pathway, we also measured concentrations of glycine and carnitine conjugates of short-to-medium chain length fatty acids in the same samples by using the same method. In addition, we quantified a spectrum of carnitine esters of 24 saturated and unsaturated fatty acids ranging from short to long chain molecules (C2–C18), including hydroxyl and dicarboxylic acids, by using tandem mass spectrometry without preceding chromatographic separation (*22*,*23*) (online Technical Appendix).

### Statistical Analyses

We compared proportions of continuous variables across groups by using the Fisher exact test and distributions of continuous variables across groups by using the Mann-Whitney U test (R version 3.2.3; R Foundation for Statistical Computing, Vienna, Austria). We performed principal component analysis for age, number of days between symptoms and disease onset, glycemia at admission, number of leukocytes in CSF, and serum levels of liver enzymes to identify grouping of characteristics that might help differentiate between infectious and toxic causes of acute encephalitis-like syndrome. We conducted principal component analysis by using Qlucore Omics Explorer software (Qlucore, Lund, Sweden).

### Ethics

Informed consent was obtained by physicians from parents of hospitalized children before sampling was conducted. The study protocol was reviewed and approved by institutional review boards at the National Institute of Hygiene and Epidemiology and the Institut Pasteur (Paris, France).

## Results

**Figure 1:**
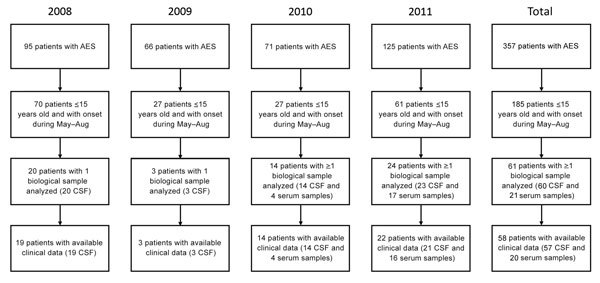
Inclusion of patients in study of hypoglycemic toxins and enteroviruses as causes of acute encephalitis-like syndrome in children, Bac Giang Province, northern Vietnam, 2008–2011. AES, acute encephalitis syndrome; CSF, cerebrospinal fluid.

A total of 185 children met the inclusion criteria over the study period (2008–2011). Median age was 5 years (interquartile range 2–8 years), and the sex ratio (male:female) was 1.4:1. The annual number of cases was higher in 2008 (70) and 2011 (61) than in 2009 (27) and 2010 (27) (Figure 1). Because of logistical constraints, CSF and blood samples were available for only 61 of the 185 children, of which 58 also had detailed clinical data. Therefore, these 58 patients represent the study population analyzed (Figure 1), including 10 patients from a previous study (*11*).

### Virologic Analyses

NGS analysis of a pool of 16 CSF samples from the 2008 outbreak provided 116,615 nonhuman contigs from 61,291,294 nonduplicated reads with an average length of 70 nt. Among these contigs, 57 with an average length of 292 nt (range 103 nt–815 nt) matched the human enterovirus B species. Fourteen contigs were assigned to the human echovirus 30 species strain Zhejiang/17/03/CSF (GenInfo Identifier DQ246620) as best hit, with nucleotide identities ranging from 83% to 98%. The second most common reference strain matched (7 contigs assigned, with best-hit ranging from 78% to 88%) was human echovirus 33 strain Toluca-3 (GenInfo Identifier 34485451). Distinct contigs mapped at same genomic locations of these 2 reference genomes and suggested that the pool of samples presumably contained >4 different virus strains. PCRs using primers designed for these contigs confirmed their sequences, identified distinct viruses in the pool, and identified patients from which the sequences had been isolated.

**Figure 2:**
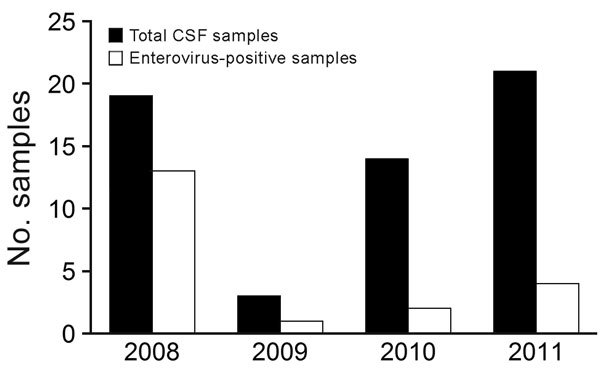
PCR-based prevalence of enterovirus infections per year in study of hypoglycemic toxins and enteroviruses as causes of acute encephalitis-like syndrome in samples (n = 57) from children, Bac Giang Province, northern Vietnam, 2008–2011. CSF, cerebrospinal fluid.

We then conducted individual NGS on 4 selected CSF samples from the pool and acquired virus genome sequences after amplification by using specifically designed PCRs. This sequencing identified 4 distinct enterovirus genomes (120486, 120492, 120488, and 120495); the first 2 genomes were closely related (Technical Appendix Figures 1–3). Prevalence of enterovirus infection was screened by PCR for CSF samples from patients with clinical data available and showed highly variable results (from 13/19 in 2008 to 4/21 in 2011) (Figure 2). Virus isolations were attempted for 10 CSF samples per annual outbreak; all showed negative results.

### Toxicologic Analysis

**Figure 3:**
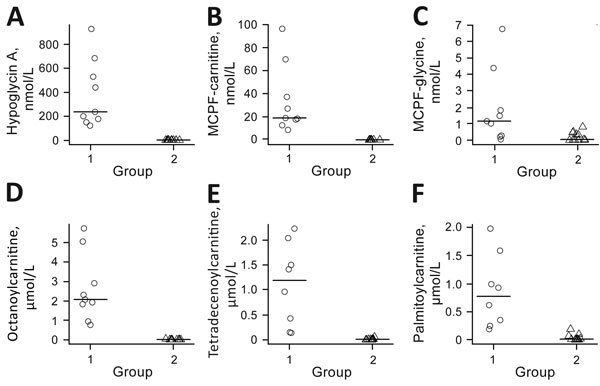
Serum concentrations of toxins and fatty acids in children with encephalitis-like syndrome, Bac Giang Province, northern Vietnam, 2008–2011. Children were grouped by high (group 1, n = 9, [circles]) and low (group 2, n = 11, [triangles]) serum concentrations of toxins. A) Hypoglycin A; B) MCPF-carnitine (methylenecyclopropylglycine metabolite); C) MCPF-glycine (methylenecyclopropylglycine metabolite); D) octanoylcarnitine (medium-chain fatty acid); E) tetradecenoylcarnitine (long-chain fatty acid in the form of acylcarnitine); and F) palmitoylcarnitine (long-chain fatty acid in the form of acylcarnitine). Horizontal lines indicate medians. MCPF, methylenecyclopropylformyl.

Although enteroviruses accounted for most (68%) cases in 2008, these viruses accounted for <20% of cases in 2009–2011. Moreover, identification of multiple and distinct enterovirus strains did not correlate with the model of an epidemic diffusion that would otherwise explain seasonal outbreaks. Therefore, we were interested in exploring alternative explanations, including 2 candidate toxins, MCPG and HGA (*13*–*15*,*17*,*18*,*24*). Twenty blood samples (4 from 2010 and 16 from 2011), which were obtained near the time of onset of symptoms, were available for this analysis. After serum analysis (Figure 3; Technical Appendix Table 2), we categorized children into 2 groups: 9 had high (>100 nmol/L) serum values of HGA and 11 had serum values of HGA below the lower limit of quantification (10 nmol/L), including 10 below the lower limit of detection (1 nmol/L).

All patients with high levels of HGA had quantifiable concentrations of methylenecyclopropylformyl (MCPF) carnitine, a metabolite of MCPG, and 6 of the 9 patients had detectable MCPF glycine, also derived from MCPG, although below the lower limit of quantification. Methylenecyclopropylacetyl conjugates, derived from HGA, were present in all of these samples, but concentrations did not reach quantifiable levels in all cases. The β-oxidation of fatty acids (Technical Appendix Table 2) was shown to be inhibited in all patients with high serum levels of HGA. As a result, concentrations of glycine and carnitine conjugates of fatty acids of short to long chain length were increased. Increased concentrations were also detected for even and odd chain length acyl compounds and unsaturated compounds in the same samples, which demonstrated complete inhibition of β-oxidation of fatty acids. Of the 9 children who had high levels of HGA/MCPG, 8 had a CSF sample tested: 1 child was positive for enteroviruses, and 7 children were negative. Of the 11 children with low levels of HGA/MCPG, 4 children were positive for enteroviruses, and 7 children were negative.

### Relationship between Epidemiologic, Clinical, and Biological Findings and Etiologies

**Figure 4:**
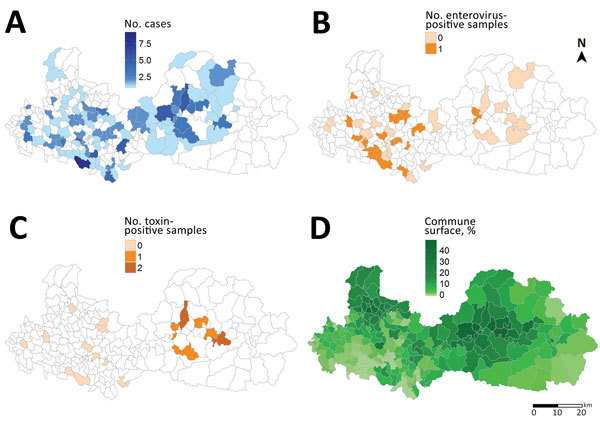
Geographic distribution of acute encephalitis-like syndrome in children, samples, and litchi cultivation at the commune level in Bac Giang Province, northern Vietnam, 2008–2011. A) No. cases of acute encephalitis-like syndrome meeting the inclusion criteria (n = 185); B) no. enterovirus-positive samples among all cerebrospinal fluid samples analyzed (n = 57); C) no. toxin-positive samples among all blood samples analyzed (n = 20); D) percentage of commune surfaces devoted to litchi cultivation.

Of the 9 children with high levels of HGA/MCPG, 8 were hospitalized in July 2011 and came from the same eastern district (Luc Ngan), a district known to have the highest levels of litchi production in Bac Giang Province (50% of province production) and in which the harvest occurs each year during June–July (Figure 4). Toxin-negative samples and enterovirus-positive samples were predominantly identified in the western part of the province.

On the basis of results of virologic and toxicologic analyses, we compared clinical and biologic characteristics among 4 patient groups: 1) the 19 children who were positive for enteroviruses and who had either low levels of HGA/MCPG (n = 4) or no blood sample tested for toxins (n = 15); 2) the 8 children with high blood levels of HGA/MCPG and who were negative for enteroviruses (n = 7) or not tested for enteroviruses (n = 1); 3) the 7 children who were negative for enteroviruses and toxins; and 4) the 23 children who were negative for enteroviruses and who were not tested for toxins (Table; Technical Appendix Table 3). One child was positive for enteroviruses and toxins and was therefore not included in statistical comparisons. All children included in the study had fever either before admission (reported by parents) or at admission, except for 1 child who had high levels of toxins, but no reported fever, who was included in the study because of severity of the neurologic condition of the child (repeated seizures and coma).

**Table:** Characteristics of 27 children hospitalized with acute encephalitis syndrome who were positive for enteroviruses or toxins, northern Vietnam, 2008–2011*

Characteristic	Enterovirus positive and toxin negative (n = 4) or not tested (n = 15)			Toxin positive and enterovirus negative (n = 7) or not tested (n = 1)		p value
	No. with data	No. (%) or median (IQR)		No. with data	No. (%) or median (IQR)	
Sex	19			8		0.80
F	NA	6 (32)		NA	3 (38)	NA
M	NA	13 (68)		NA	5 (62)	NA
Age, y	19			8		0.47
<2	NA	4 (21)		NA	3 (38)	NA
2–4	NA	5 (26)		NA	3 (38)	NA
5–9	NA	7 (37)		NA	1 (12)	NA
10–15	NA	3 (16)		NA	1 (12)	NA
Symptoms/signs before and at admission						
Temperature at admission, °C	18	38.0 (37.6–38.5)		7	37.5 (37.4–37.8)	0.14
Fever before admission	18	18 (100)		7	5 (71)	0.07
Headache	19	11 (58)		6	3 (50)	1.0
Seizures	18	5 (28)		8	7 (88)	0.009
Coma	14	4 (29)		7	4 (57)	0.35
Meningeal symptoms	18	12 (67)		7	4 (57)	0.67
Limb paralysis	18	1 (6)		5	0	1.0
Vomiting	18	14 (78)		7	6 (86)	1.0
Diarrhea	14	3 (21)		6	0	0.52
Days from disease onset to admission	19	2.0 (0.5–2.5)		8	0.0 (0.0–0.2)	0.008
Blood sample						
Leukocytes, × 10^9^ cells/L	18	10.5 (7.5–15.8)		8	19.5 (18.4–29.9)	0.004
Platelets/μL	12	254 (197–306)		7	340 (274–487)	0.20
Hemoglobin, g/L	7	114 (108–118)		6	116 (92–122)	0.77
Glucose, mmol/L	14	4.5 (3.9–5.0)		7	2.0 (1.6–3.8)	0.67
Glucose <3 mmol/L	14	0		7	5 (71)	0.001
Cerebrospinal fluid sample						
Leukocytes/mm^3^	12	50 (6–100)		6	3 (1–3)	0.001
Lymphocytes/mm^3^	8	80 (73–80)		1	45 (45–45)	0.31
Protein level >0.5 g/L	15	4 (27)		6	0	0.28
Transparent appearance of CSF	15	15 (100)		7	7 (100)	1.0
Liver enzymes at or after admission, IU/L						
Alanine aminotransferase	8	24 (12–33)		8	48 (37–56)	0.04
Aspartate aminotransferase	8	28 (20–46)		8	68 (62–79)	0.01

*CSF, cerebrospinal fluid; IQR, interquartile range; NA, not applicable.

Children with high blood levels of HGA/MCPG had shorter median time between disease onset and admission (0 days vs. 2 days; p = 0.008), more seizures (88% vs. 28%; p = 0.009), more hypoglycemia (glucose level <3 mmol/L) (71% vs. 0%; p = 0.001), lower median numbers of leukocytes in CSF (3 cells/mm^3^ vs. 50 cells/mm^3^; p = 0.001), and higher median serum levels of alanine aminotransferase (48 IU/L vs. 24 IU/L; p = 0.04) and aspartate aminotransferase (68 IU/L vs. 28 IU/L; p = 0.01) than patients infected with enteroviruses. Two (25%) of 8 children who were positive for toxins died, whereas only 1 (5.3%) of the 19 children with enterovirus encephalitis died, but this difference was not significant (p>0.05).

Principal component analysis showed that these children with high levels of HGA/MCPG clustered differently in the projection space (Technical Appendix Figure 4) than children with evidence of infection with enteroviruses (Technical Appendix Table 4). Furthermore, children not infected with enteroviruses for whom HGA/MCPG showed negative results or was not tested had profiles more similar to children infected with enteroviruses. In particular, these children had glycemia (glucose level >3 mol/L), and leukocyte counts in CSF were slightly increased (Technical Appendix Table 3). 

## Discussion

This study suggests that acute encephalitis-like syndrome previously associated spatially and temporally with litchi harvests (*11*) was caused by intoxication, rather than viral encephalitis, as initially suspected. In this context of recurrent acute encephalitis-like syndrome outbreaks since 1999, no consistent viral etiology had been identified by using standard laboratory diagnostic techniques. Because of the high number of viruses known to be associated with encephalitis (*3*,*5*,*25*), we used NGS to analyze samples of patients after the annual outbreak in 2008. This hypothesis-free technique identified human enterovirus B serotypes that were further confirmed by PCR. Other pathogens were not identified.

Despite the link shown in this study between several acute encephalitis-like syndrome cases and enteroviruses, the frequency of enterovirus infection among clinical cases was highly variable from year to year (Figure 2). Therefore, we tested serum samples of a subset of enterovirus-negative and enterovirus-positive cases for HGA and MCPG metabolites. Of 20 children tested, 9 (45%) were positive for HGA and MCPG metabolites and 8 showed inhibition of the β-oxidation catabolic process. Eight of these 9 case-patients came from the same district, which is known for having the highest litchi production in the province. These case-patients were hospitalized in July 2011, at the time of the litchi harvest in the district. These 8 case-patients appeared to have distinct characteristics in comparison with those who had enterovirus acute encephalitis syndrome, including younger age, more rapid progression, higher frequency of seizures, severe hypoglycemia, lack of increased numbers of leukocytes in CSF, and moderate increases in levels of liver enzymes.

The clinical and biochemical presentation of these case-patients clearly matches that of case-patients reported during outbreaks linked to litchi harvests in India and Bangladesh (*12*,*15*,*16*) and of case-patients with Jamaican vomiting sickness (*24*). Investigation of an outbreak in Muzaffarpur, India, recently concluded that intoxication with HGA and MCPG was responsible for the outbreak, a finding that is consistent with our results (*26*), which also identified HGA/MCPG in young patients with hypoglycemic encephalopathy. Our study also provides a direct comparison of clinical and biologic profiles of acute encephalitis-like syndrome related to enterovirus infection versus intoxication with HGA/MCPG. Thus, we provide useful information that can be used to guide clinical decision making, particularly the need for glycemia testing for management of patients with acute encephalitis-like syndrome.

Our study was limited by comparison of children subjected to different tests at different times (only blood samples obtained during 2010 and 2011 were available for testing of toxins, whereas testing for enterovirus was available throughout the study) and by having used fever as an inclusion criteria. In the study by Shrivastava et al. in India, in which this inclusion criterion was not used, 61% of children with litchi intoxication were afebrile (*26*). As a result of using fever as an inclusion criteria, our study might have missed several children with HGA/MCPG intoxication. Apart from 1 patient who had enterovirus infection and high serum concentrations of hypoglycemic toxins, patients with enterovirus infections did not have higher toxin levels than patients without enterovirus infections, suggesting that subtoxic concentrations of HGA/MCPG were not associated with increased risk for enterovirus infection. However, studies with larger numbers of patients are needed to rule out this hypothesis.

We also tried to elucidate the link between these 2 etiologies (enteroviruses and hypoglycemic toxins) and litchi harvesting in northern Vietnam. For enteroviruses, it is likely that temperature and humidity conditions required for enterovirus circulation match those of litchi maturation and harvest. For intoxication with HGA and MCPG, the link is more obvious because toxins have been previously identified in the litchi aril and litchi seeds (*17*,*18*). In our study, cases of intoxication clustered geographically in areas of large production of litchi. Levels of hypoglycemic amino acids in the litchis are not known. Results from 2 studies suggest that MCPG concentration is highest in the seeds, followed by arils of semiripe litchis and then ripe litchis (*17*,*26*). Further investigations should compare levels of toxins across cultivars and soil, climate, and harvest conditions, as recommended by Spencer et al. (*27*). To further investigate a causal link between HGA/MCPG levels and acute encephalitis-like syndrome, healthy children exposed to the same litchi intake would need to be tested. Nevertheless, the evidence of inhibited β-oxidation of fatty acids in all HGA/MCPG–positive patients in this study is a convincing demonstration that intoxication was a key driver of symptoms in these patients.

Intoxication with HGA/MCPG is attributed mainly to a hypoglycemic encephalopathy, secondary to inhibition of β-oxidation and an inability to produce glucose from fatty acids. This metabolic process usually takes hours, which might explain why most children have initial symptoms during the second half of the night. Shrivastava et al. reported that children who had no evening meal were at higher risk for developing hypoglycemic encephalopathy (*26*). Young children, and even more so undernourished children, have limited glycogen stores, which increases their vulnerability to the effects of intoxication with HGA/MCPG on metabolism (*13*,*15*). Concentrations of glycine and carnitine conjugates measured in serum samples might appear rather low. These conjugates would be better measured in urine samples (*28*). However, such samples were not available in this study.

In conclusion, this study has shown that within a context of largely viral encephalitis, particularly encephalitis caused by enteroviruses, acute hypoglycemic encephalopathy developed in some children in Vietnam during the litchi harvest, possibly after absorption of a toxin present in the aril of litchi fruits. Local populations should be sensitized to the risks associated with young children eating litchis. Also, for children coming to healthcare facilities because of acute encephalitis-like syndrome during the litchi harvest season, measurement of blood glucose concentrations and immediate infusion with dextrose for those children with hypoglycemia should be critical elements of clinical management. Use of these elements will likely increase patient survival.

Technical Appendix Additional information on hypoglycemic toxins and enteroviruses as causes of acute encephalitis-like syndrome in children, Bac Giang Province, northern Vietnam.
