# Supplementary material for: Hypoglycemic Toxins and Enteroviruses as Causes of Outbreaks of Acute Encephalitis-Like Syndrome in Children, Bac Giang Province, Northern Vietnam
Source: Emerg Infect Dis. 2018 Aug;24(8):1435–43. doi: 10.3201/eid2408.171004 (PMC6056107; doi:10.3201/eid2408.171004)
Supplement: Technical Appendix — Additional information on hypoglycemic toxins and enteroviruses as causes of acute encephalitis-like syndrome in children, Bac Giang Province, northern Vietnam. [file 17-1004-Techapp-s1.pdf]

Article DOI: https://doi.org/10.3201/eid2408.171004

Hypoglycemic Toxins and Enteroviruses as Causes of Acute Encephalitis-Like Syndrome in Children, Bac Giang Province, Northern Vietnam

Technical Appendix

Materials and Methods

Virus Isolation

Whenever sufficient material was available, we inoculated cerebrospinal fluid (CSF) samples onto RD, Vero E6, or C6/36 monolayers. Cell cultures were checked daily for eventual cytopathic effects and harvested 7 days after inoculation. Viral replication was tested by using reverse transcription PCR (RT-PCR).

Purification of Nucleic Acids and Random Amplification

Nucleic acids were purified from 150 µL of CSF by using the NucleoSpinDxVirus Kit (Macherey-Nagel GmbH and Co. KG, Düren, Germany), according to the manufacturer’s instructions. Purified nucleic acids were eluted in 60 µL of buffer. Eight microliters of purified nucleic acids (containing RNA) was reverse transcribed by using the First-Strand Synthesis Superscript III Kit (Invitrogen, Carlsbad, CA, USA). Complementary DNA and DNA were then amplified by using the Whole Transcriptome Amplification Kit (QIAGEN, Hilden, Germany), according to the manufacturer’s instructions. Resulting products were used undiluted or diluted up to 1,000 times in nuclease-free ultrapure water before use in preparation of libraries for high-throughput sequencing (HTS) or as a template in specific PCRs (*1*–*5*) (Technical Appendix Table 1).

Virus Detection by RT-PCR

CSF samples were screened by using RT-PCR for viruses already associated with acute encephalitis syndrome. In brief, we tested for herpes simplex virus, measles virus, varicella zoster

virus, enterovirus, dengue virus, and Nipah virus. Alternatively, because of limited amounts of samples, PCR were performed on whole transcriptome amplification products. Data obtained from HTS of pooled samples from the outbreak in 2008 enabled us to design specific RT-PCRs for screening future outbreaks (2008–2011). Conditions for amplification using in-house primers (Technical Appendix Table 1) were at 95°C for 2 min; followed by 35 cycles at 95°C for 30 s, 55°C for 30 s, and 72°C for 2 min; and a final extension step at 72°C for 5 min. These in-house PCRs were complemented by the use of a PCR specific for conserved regions of enterovirus genomes (*1–5*) (Technical Appendix Table 1) and the Argene Enterovirus Detection Kit (bioMérieux, Marcy l'Etoile, France).

Preparation for HTS

The strategy was to explore the metagenome of 16 CSF samples from patients matching the case definition for acute encephalitis syndrome during the outbreak in 2008. A total of 0.2 μg of nucleic acid from each amplified product was pooled and analyzed by using HTS. The 400-nt library was prepared from the pool by using the TruSeq DNA Kit (Illumina, San Diego, CA, USA) and paired-end sequenced by using an HiSeq 2000 Apparatus (Illumina). PCRs designed for contigs sequenced from first-round HTS data enabled selection of 4 CSFs that were individually deep sequenced by using the same method to obtain larger genomic sequences.

HTS and Raw Data Processing

Sequencing was performed on a half-lane of a HiSeq 2000 sequencer for each CSF sample. Raw data (70Mio reads for each CSF) processing consisted of 3 successive filtering steps: duplicated reads removal; quality trimming according to the Phred score quality (minimum of 15 at 3′ and 5′, minimal length after trimming set to 30 nt); and host removal (Soap2 mapping against hg18) (*6*). Remaining reads (31Mio reads) were assembled by using CLC de novo assembly software (CLCBio; QIAGEN). Contigs were aligned against whole nucleic acid and protein databases (https://www.ncbi.nlm.nih.gov/) by using Smith-Waterman algorithm (default parameters but with mismatch penalty set to –1). Taxonomic assignation was performed by using a best-hit strategy; the first alignment was considered and the taxonomic identifier was selected.

HTS Alignment on a Reference Strategy

The best-hit strategy enabled us to select the closest genome reference for a specific taxonomic identifier. All generated contigs were aligned to these references by using a local Smith-Waterman alignment. Relevant alignments have been selected to design PCR primers for Sanger sequencing. In

addition, a mapping strategy (Soap2 aligner) was used to produce coverage and depth of sequencing statistics for each complete genome.

PCR and Sanger Sequencing

PCR products were obtained and sequenced by using in-house–designed primer pairs and pan-enterovirus primer sets (*1*,*3*–*5*). PCR amplifications were performed by using Taq DNA polymerase (Invitrogen). Conditions for amplification by using in-house primers are detailed above. Nucleotide sequences were obtained by using Big Dye Version 1.1 chemical analysis on an ABI 3730XL Apparatus (Applied Biosystems, Foster City, CA, USA).

Genome Analysis and Phylogenetics

Presence of open reading frames in sequenced genomes was investigated by using CLC 4 Main Workbench software version 6.8.4 (QIAGEN). Closest similarities with known enteroviruses were determined by using the BLAST algorithm on viral protein 1 (VP1) and other major open reading frames (*7*). The position and likelihood of recombinations were evaluated by a number of recombination detection algorithms (RDP, geneconv, Bootscan, Maxchi, Chimera, SIScan) by using the RDP3 package (*8*). Using the same complete genomes database, we inferred a VP1-based phylogeny of enteroviruses by using sequences detected in this study and those of 82 serotypes of Human Enterovirus B available on public databases. The following sequences were used: AF326751, HM777023, X77708, AY302542, AY302540, AY302549, AY302539, AY302551, AY302550, EF174469, AF081485, AY843302, AY843297, AY843300, JQ041368, AF105342, DQ480420, X05690, AY843299, EF066392, AY896767, AY302553, E6U16283, AF268065, AY429470, AF230973, AF114383, JN695051, AY896761, JQ729993, AF524866, X84981, CXA9CG, AY302547, EF371880, AF465516, AY896763, GU109481, NC001472, CXA1G, AY843301, AY843298, AY302557, AY302543, CXA3G, AF241359, AY302552, AY302546, AY302556, AY302541, AY302555, AY302544, AY302560, AY302548, AF465518, AY302554, HM775882, AF083069, AY843307, AY896765, AY843304, AJ577594, AY556070, AY556057, AY896764, NC009887, NC013115, AY843303, NC013114, AY843305, AY843306, AY843308, DQ902712, HM777023, AM236984, FJ868349, AM492410, HQ897643, and FJ525936.

Sequences were first aligned by using Muscle software (*9*). The CLC main workbench 6.8.4 and Mega5 software were also used to manipulate the sequences and alignments (*10*). The Bayesian Information Criterion, the corrected Akaike Information Criterion scores, and maximum-likelihood

values were calculated by using jModeltest software (*11*). A Bayesian model was set up and tested by using BEAST (*12*).

According to corrected Akaike Information Criterion estimations, the most appropriate pattern of substitutions was as previously used and was complemented by a discrete gamma distribution of evolutionary rates among sites, assuming that certain sites remained invariable. An uncorrelated lognormal distribution with a default uniform prior ranging from 0 to 1 was used to model the rate of evolution. Several coalescent based models were tested as tree priors. The Markov chain Monte Carlo was set to 10,000,000 states, and 10,000 trees were sampled to obtain an adequate posterior effective sample size >200.

Identification of Recombinants

Virus genomes were included in recombination detection tests and molecular typing approaches. Phylogenetic analysis showed clustering of VP1 sequences from each sample with respective serotypes of human enterovirus B previously identified by the BLAST analysis (Technical Appendix Figure 1). Other parts of the genome, such as genes 2 (A, B, C) and 3 (A, C, D), clustered differently than VP1 and had robust statistical support, indicating that enteroviruses from 2008 were potential recombinants (Technical Appendix Figures 2, 3). Specific analyses of recombination statistically supported several putative recombination breakpoints (Technical Appendix Figure 2).

Interpretation

Among human enterovirus B serotypes known to be associated with encephalitis (*13*), E6, E9, E16, E30, and E3 were reported in this study. Serotypes E6, E30, and E16 have been reported as a cause of meningitis and, more recently, human enterovirus B (CVA9, CVB1–5, E3–7, E9, E11, E13, E14, E16–19, E24, E25, E27, E30, and E33) have been associated with encephalitis in China (*14*,*15*). The serotypes detected in our study are present in recombinant viruses (Technical Appendix Figures 2, 3). The severe clinical presentation observed with recombinant serotypes circulating during outbreaks might be related to a putative role of recombination in pathogenesis, as proposed by Lukashev et al. for E30 (*16*). The recombinants described in our study might result from intense cocirculation of diverse enteroviruses in an ecologic context that might favor emergence of new variants of neurovirulent human enterovirus B.

Toxicologic Analysis

Blood samples were tested for hypoglycin A (HGA), carnitine and glycine conjugates of methylenecyclopropylacetyl (metabolite of HGA) and methylenecyclopropylformyl (metabolite of

methylenecyclopropylglycine [MCPG]), and short-to-medium length chain fatty acids by using a modification of a reported analytical method (*17*,*18*). Sample volume was increased and dilution of extracts was reduced to increase sensitivity. In brief, a methanolic internal standard solution (300 µL) was added to 25 µL of serum or urine for extraction, and the mixture was vortexed for 20 s and centrifuged for 10 min at a relative centrifugal force (RCF) of 17,000. From the clear supernatant, 250 µL was removed and dried in a microtiter plate at 65°C for ≈30 min under a gentle stream of nitrogen. The residue was treated with 50 µL of 3N butanol-HCl for 15 min at 65°C and dried again at 65°C under nitrogen. The dry material was dissolved in in 70 µL of methanol:water (80:20 vol/vol) and further diluted 1:2 with water. From this solution 90 µL was transferred to a 384 microtiter plate, centrifuged at an RCF of 17,000 to sediment any particles, and then used for ultraperformance liquid chromatography–tandem mass spectrometry. From this solution, 5 µL was injected onto an ACQUITY UPLC BEH C18 1.7 µm, 2.1 × 50 mm column (Waters, Eschborn, Germany) for gradient chromatography. Tandem mass spectrometric analysis was performed with single-point calibration on a Xevo TQ-MS UPLC-MS/MS System (Waters). The lower limit of detection for all compounds was ≈1 nmol/L, and the lower limit of quantification was 10 nmol/L.

In addition, a spectrum of 24 carnitine esters of saturated and unsaturated fatty acids ranging from short-chain to long-chain molecules (C2–C18), including hydroxy and dicarboxylic acids, was quantified by using tandem mass spectrometry without preceding chromatographic separation, according to standard methods used in newborn screening for inborn errors of metabolism (*19*,*20*). Serum was diluted 1:5 with water, and 17.5 µL of this solution were extracted with 100 µL of methanol containing 13 deuterized internal standards. After centrifugation at an RCF of 17,000, the supernatant was transferred to a microtitration plate and dried under a gentle stream of nitrogen at 65°C. Butylation of the carnitine esters of fatty acids and of free carnitine was performed by adding 50 µL of HCl-butanol and incubating for 15 min at 65°C. After drying, the residue was dissolved in 200 µL of methanol/water (80:20 vol/vol). From this solution, 20 µL was injected directly onto the electrospray ionization interface of a tandem mass spectrometer (Quattro Micro, Waters, Eschborn, Germany). Concentrations of acyl carnitines were deduced by comparing peak height to those of internal standards.

Principal Component Analysis

Methods

To study clinical and biologic profiles of the 58 patients, principal component analysis (PCA) was performed for age, temperature at admission, number of days between disease onset and symptoms, glycemia at admission, number of leukocytes in CSF, and blood levels of liver enzymes (aspartate aminotransferase and alanine aminotransferase). We determined which groups of patients were similar or different and characterized these groups by variables or groups of variables. Variables were normalized before performing PCA. We excluded from the analysis 1 person who had an excessive contribution to the first principal component (exceeding its weight), which could have caused unreliable results.

Results

We retained the first 3 principal components, which had eigenvalues >1, indicating that they accounted for more variance than accounted by 1 of the original variables. Together, they accounted for 68% of total variance.

Interpretation of Principal Components

We determined correlation coefficients between initial variables and the 3 principal components (Technical Appendix Table 3). For interpretation of PCA, coefficients of interest are those whose absolute value is closer to 1.

The first principal component was negatively correlated with levels of aspartate aminotransferase and alanine aminotransferase and positively correlated with glycemia, thus differentiating children with standard levels of liver enzymes and glucose from children with signs of intoxication (higher levels of liver enzymes and lower levels of glucose). The second principal component was positively correlated with delay between disease onset and admission and leukocytes in CSF, thus differentiating children with higher levels of leukocytes (sign of infection) and less rapid progression of the disease from children with standard levels of leukocytes and a more rapid disease progression. The third principal component was negatively correlated with age, thus differentiating younger children from older children.

Three-Dimensional Representation of Patients

We obtained a projection of patients in 3-dimensional space formed by principal components (Technical Appendix Figure 4). Children with higher levels of HGA/MCPG (group 2) formed a distinct cluster in the projection space that differed from children infected with enteroviruses (group 1). Children with higher levels of HGA/MCPG had increased levels of liver enzymes and more severe hypoglycemia (negative coordinates on the first axis), more rapid progression of the disease and standard levels of leukocytes in CSF (negative coordinates on the second axis), and a younger age (positive coordinates on the third axis). Children not infected with enteroviruses for whom HGA/MCPG levels were low (group 3) or not tested (group 4) had profiles more similar to those with enteroviruses.

References

1. Boros A, Pankovics P, Knowles NJ, Reuter G. Natural interspecies recombinant bovine/porcine enterovirus in sheep. J Gen Virol. 2012;93:1941–51. PubMed http://dx.doi.org/10.1099/vir.0.041335-0

2. Zoll GJ, Melchers WJ, Kopecka H, Jambroes G, van der Poel HJ, Galama JM. General primer-mediated polymerase chain reaction for detection of enteroviruses: application for diagnostic routine and persistent infections. J Clin Microbiol. 1992;30:160–5. PubMed

3. Puig M, Jofre J, Lucena F, Allard A, Wadell G, Girones R. Detection of adenoviruses and enteroviruses in polluted waters by nested PCR amplification. Appl Environ Microbiol. 1994;60:2963–70. PubMed

4. Oberste MS, Maher K, Kilpatrick DR, Flemister MR, Brown BA, Pallansch MA. Typing of human enteroviruses by partial sequencing of VP1. J Clin Microbiol. 1999;37:1288–93. PubMed

5. Nix WA, Oberste MS, Pallansch MA. Sensitive, seminested PCR amplification of VP1 sequences for direct identification of all enterovirus serotypes from original clinical specimens. J Clin Microbiol. 2006;44:2698–704. PubMed http://dx.doi.org/10.1128/JCM.00542-06

6. Li R, Yu C, Li Y, Lam T-W, Yiu S-M, Kristiansen K, et al. SOAP2: an improved ultrafast tool for short read alignment. Bioinformatics. 2009;25:1966–7. PubMed http://dx.doi.org/10.1093/bioinformatics/btp336

7. Altschul SF, Gish W, Miller W, Myers EW, Lipman DJ. Basic local alignment search tool. J Mol Biol. 1990;215:403–10. PubMed http://dx.doi.org/10.1016/S0022-2836(05)80360-2

8. Martin DP, Lemey P, Lott M, Moulton V, Posada D, Lefeuvre P. RDP3: a flexible and fast computer program for analyzing recombination. Bioinformatics. 2010;26:2462–3. PubMed http://dx.doi.org/10.1093/bioinformatics/btq467

9. Edgar RC. MUSCLE: multiple sequence alignment with high accuracy and high throughput. Nucleic Acids Res. 2004;32:1792–7. PubMed http://dx.doi.org/10.1093/nar/gkh340

10. Tamura K, Peterson D, Peterson N, Stecher G, Nei M, Kumar S. MEGA5: molecular evolutionary genetics analysis using maximum likelihood, evolutionary distance, and maximum parsimony methods. Mol Biol Evol. 2011;28:2731–9. PubMed http://dx.doi.org/10.1093/molbev/msr121

11. Posada D. jModelTest: phylogenetic model averaging. Mol Biol Evol. 2008;25:1253–6. PubMed http://dx.doi.org/10.1093/molbev/msn083

12. Drummond AJ, Rambaut A. BEAST: Bayesian evolutionary analysis by sampling trees. BMC Evol Biol. 2007;7:214–214. PubMed http://dx.doi.org/10.1186/1471-2148-7-214

13. Tapparel C, Siegrist F, Petty TJ, Kaiser L. Picornavirus and enterovirus diversity with associated human diseases. Infect Genet Evol. 2013;14:282–93. PubMed http://dx.doi.org/10.1016/j.meegid.2012.10.016

14. Zhao YN, Jiang QW, Jiang RJ, Chen L, Perlin DS. Echovirus 30, Jiangsu Province, China. Emerg Infect Dis. 2005;11:562–7. PubMed http://dx.doi.org/10.3201/eid1104.040995

15. Zhang L, Yan J, Ojcius DM, Lv H, Miao Z, Chen Y, et al. Novel and predominant pathogen responsible for the enterovirus-associated encephalitis in eastern China. PLoS One. 2013;8:e85023. PubMed http://dx.doi.org/10.1371/journal.pone.0085023

16. Lukashev AN, Ivanova OE, Eremeeva TP, Gmyl LV. Analysis of echovirus 30 isolates from Russia and new independent states revealing frequent recombination and reemergence of ancient lineages. J Clin Microbiol. 2008;46:665–70. PubMed http://dx.doi.org/10.1128/JCM.02386-06

17. Sander J, Terhardt M, Sander S, Janzen N. Quantification of hypoglycin A as butyl ester. J Chromatogr B Analyt Technol Biomed Life Sci. 2016;1029-1030:169–73. PubMed http://dx.doi.org/10.1016/j.jchromb.2016.07.005

18. Sander J, Cavalleri J-MV, Terhardt M, Bochnia M, Zeyner A, Zuraw A, et al. Rapid diagnosis of hypoglycin A intoxication in atypical myopathy of horses. J Vet Diagn Invest. 2016;28:98–104. PubMed http://dx.doi.org/10.1177/1040638715624736

19. Chace DH, DiPerna JC, Naylor EW. Laboratory integration and utilization of tandem mass spectrometry in neonatal screening: a model for clinical mass spectrometry in the next millennium. Acta Paediatr Suppl. 1999;88:45–7. PubMed http://dx.doi.org/10.1111/j.1651-2227.1999.tb01156.x

20. Rashed MS, Rahbeeni Z, Ozand PT. Application of electrospray tandem mass spectrometry to neonatal screening. Semin Perinatol. 1999;23:183–93. PubMed http://dx.doi.org/10.1016/S0146-0005(99)80050-0

Technical Appendix Table 1. Primers used for study of hypoglycemic toxins and enteroviruses as causes of acute encephalitis-like syndrome in children, Bac Giang Province, northern Vietnam, 2008–2011*

Primer	Gene	Sequence, 5′→3′	Reference
CoxB2/ctig74058+	3B	AGACAGGAGGAACCAGGTGAG	This study
CoxB2/ctig74058-	3B	GAAACAGTGCCTCAAGGGTGG	This study
CoxB3/ctig73505+	2B/2C	GGCTCAAGGTCAAGATCTTACC	This study
CoxB3/ctig73505-	2B/2C	GGGAAAATACTCTCTTGGCCTC	This study
CoxB5/ctig72615+	2B/2C	AGATCTTGCCAGAGGTGAAGG	This study
CoxB5/ctig72615-	2B/2C	AGTTGTTCCTGATCGCTCTGG	This study
CoxB6/ctig1095+	2A	TGCCAATGCACAATGGGGCTG	This study
CoxB6/ctig1095-	2A	CCCATAGTCACGAGTCCGATG	This study
Echo4/ctig51733+	3B/3C	AAGAGATGCTGCCCCCTTGTG	This study
Echo4/ctig51733-	3B/3C	CCTTAGCGTGGGCACTTTAGG	This study
Echo5/ctig108676+	3B/3C	TGCATACACAGGACTGCCCAA	This study
Echo5/ctig108676-	3B/3C	CTTCACTGTGCTGGAATTCCTC	This study
Echo6/ctig2864+	VP2/VP3	CGCCGAGTACAACGGATTGAG	This study
Echo6/ctig2864-	VP2/VP3	AACCAAAGACCTGCGACCCAG	This study
Echo7/ctig7637+	3D	TTCTGGGACTAGCATCTTCAAC	This study
Echo7/ctig7637-	3D	TCATAGGCATAACGGGATGAAC	This study
Echo9/ctig114121+	VP4/VP2	AGTTGCGCAGTGTTTCGCTCC	This study
Echo9/ctig114121-	VP4/VP2	GTGTTCAATGCCGCGTAGTCC	This study
Echo17/ctig26838+	2B/2C	AGTGAACGCCCAATCAGGTTAG	This study
Echo17/ctig26838-	2B/2C	CCGTATTGAGCCCGTATGTTTG	This study
Echo20/ctig90216+	3D	CACTGTTTCAAGGTCCACCAG	This study
Echo20/ctig90216-	3D	ACGCCTGGAGACAAATGACAG	This study
Echo24/ctig98651+	3B/3C	CAACACGGCCTCGTTAACTTC	This study
Echo24/ctig98651-	3B/3C	TTGTCTCCAGGCCCTAACTAC	This study
Echo25/ctig93383+	2C/3A	CTCCCTACCACCTGATCCAC	This study
Echo25/ctig93383-	2C/3A	CCTTCTCTTCTAGCGCAGCC	This study
Echo33/ctig50620+	VP1	CCTGGATCAGTCAGACGCAC	This study
Echo33/ctig50620-	VP1	ACTGGAGCATCCTGGCTCAC	This study
Echo79/ctig65990+	2B	ATGGGTGGCTAAAGAAATTTACTG	This study
Echo79/ctig65990-	2B	TTGAGCCACTCAATAAACTTCTG	This study
UnivEntero-5′ UTR-F	5′ UTR	GTACCYTTGTRCGCCTGTT	(*1*)
UnivEntero-5′ UTR-R	5′ UTR	ATTGTCACCATAAGCAGCCA	(*2*)
Ent1	5′ UTR	CGGTACCTTTGTACGCCTGT	(*3*)
Ent2	5′ UTR	ATTGTCACCATAAGCAGCCA	(*3*)
EV-040	VP1/2A	ATGTAYRTICCIMCIGGIGC	(*4*)
EV-011	VP1/2A	GCICCIGAYTGITGICCRAA	(*4*)
224	VP3	GCIATGYTIGGIACICAYRT	(*5*)
222	VP1	CICCIGGIGGIAYRWACAT	(*5*)
AN89	VP1	CCAGCACTGACAGCAGYNGARAYNGG	(*5*)
AN88	VP1	TACTGGACCACCTGGNGGNAYRWACAT	(*5*)

*Cox, coxsackievirus; ctig, contiguous; Echo, echovirus; Ent, enterovirus; EV, enterovirus; F, forward; R, reverse; UnivEntero, universal enterovirus; UTR, untranslated region; VP, virus capsid protein.

Technical Appendix Table 2. Serum concentrations of HGA and its metabolites, MCPG metabolites, and short-to-medium chain and long chain fatty acids in children with acute encephalitis syndrome, Bac Giang Province, northern Vietnam, 2010–2011*

Sample ID	Group	Hypoglycin A, nmol/L	MCPA-carnitine, nmol/L	MCPA-glycine, nmol/L	MCPF-carnitine, nmol/L	MCPF-glycine, nmol/L	Butyryl-carnitine, µmol/L	Isovalery-carnitine, µmol/L	Octanoyl-carnitine, µmol/L	Dodecanoyl-carnitine, µmol/L	Tetradecenoyl-carnitine, µmol/L	Palmitoyl-carnitine, µmol/L	Stearoyl-carnitine, µmol/L	Oleyl-carnitine, µmol/L
c1	Control	0.03	0.00	0.24	0.05	0.95	0.12	0.05	0.09	0.09	0.11	0.16	0.09	0.18
c2	Control	0.01	0.00	0.37	0.04	0.03	0.12	0.05	0.09	0.08	0.09	0.15	0.11	0.18
c3	Control	0.01	0.00	0.38	0.05	0.06	0.11	0.05	0.09	0.07	0.11	0.15	0.01	0.16
40	1	928.60	6.85	24.06	37.27	1.03	3.79	1.69	2.91	1.43	2.05	1.98	0.32	1.27
36	1	685.19	14.47	32.85	69.92	1.49	6.37	2.34	5.73	1.40	1.51	0.99	0.31	0.82
44	1	529.39	1.32	26.76	17.78	6.78	1.32	0.65	2.31	1.14	2.24	1.59	0.27	1.35
46†	1	440.14	12.97	55.64	96.31	1.83	7.60	4.63	5.06	ND	ND	ND	ND	ND
48	1	236.20	1.27	16.81	18.58	4.40	2.73	1.21	2.07	0.54	0.97	0.93	0.17	0.86
42	1	200.62	1.40	5.32	27.18	1.16	1.87	0.54	1.93	0.60	0.43	0.35	0.09	0.30
16	1	177.16	1.31	15.57	18.98	0.28	4.18	0.32	1.84	0.15	0.15	0.25	0.10	0.41
38	1	149.00	0.16	0.89	8.76	0.05	0.60	0.16	0.77	0.14	0.14	0.19	0.08	0.20
52	1	121.38	1.92	3.16	12.75	0.21	1.24	3.92	0.95	1.50	1.42	0.62	0.27	0.36
34	2	3.15	0.00	0.46	0.20	0.51	0.03	0.01	0.01	0.01	0.00	0.01	0.01	0.00
30	2	0.99	0.00	0.35	0.26	0.00	0.08	0.01	0.01	0.00	0.00	0.00	0.00	0.00
18	2	0.25	0.00	0.33	0.18	0.36	0.17	0.05	0.05	0.05	0.06	0.19	0.11	0.26
32	2	0.18	0.00	0.31	0.22	0.01	0.16	0.05	0.02	0.01	0.01	0.01	0.01	0.01
12	2	0.17	0.02	0.41	0.14	0.04	0.34	0.02	0.01	0.03	0.01	0.10	0.05	0.08
14	2	0.17	0.00	0.27	0.03	0.46	0.14	0.08	0.01	0.01	0.01	0.07	0.03	0.06
24	2	0.04	0.00	0.36	0.06	0.02	0.11	0.02	0.01	0.01	0.01	0.00	0.01	0.00
22	2	0.03	0.00	0.34	0.14	0.02	0.19	0.11	0.02	0.02	0.01	0.01	0.00	0.01
26	2	0.02	0.01	0.35	0.01	0.81	0.08	0.03	0.01	0.01	0.00	0.01	0.00	0.01
20	2	0.01	0.01	0.32	0.23	0.00	0.06	0.03	0.01	0.01	0.00	0.01	0.00	0.00
28	2	0.01	0.00	0.36	0.15	0.32	0.11	0.10	0.01	0.03	0.01	0.02	0.01	0.01
Group ratio		1,402.27	705.40	48.63	123.91	26.07	24.36	36.79	172.08	87.00	119.50	77.50	22.00	61.50

*Group ratio is the median of group 1 divided by the median of group 2. Children were divided into 2 groups: 9 had high (>100 nmol/L) values of HGA in serum (group 1), and 11 (group 2) had values below the limit of quantification (10 nmol/L), including 10 below the limit of detection (1 nmol/L) (group 2). Samples were sorted by decreasing order of HGA concentrations. Samples c1, c2, and c3 were from a healthy adult. HGA, hypoglycin A; ID, identification; MCPA, methylenecyclopropylacetyl; MCPF, methylenecyclopropylformy; MCPG, methylenecyclopropylglycine; ND, not determined.
†Ultraperformance liquid chromatography–tandem mass spectrometry was performed with 40% of the serum volume.

Technical Appendix Table 3. Characteristics of 30 hospitalized children with acute encephalitis syndrome, Bac Giang Province, northern Vietnam, 2008–2011*

	Enterovirus negative, toxin negative (n = 7)		Enterovirus negative, toxin not tested (n = 23)	
Characteristic	No. with data	No. (%) or median (IQR)	No. with data	No. (%) or median (IQR)
Sex	7	NA	23	NA
F	NA	4 (57)	NA	12 (52)
M	NA	3 (43)	NA	11 (48)
Age, y	7	NA	23	
<2	NA	1 (14)	NA	5 (22)
2–4	NA	1 (14)	NA	6 (26)
5–9	NA	4 (57)	NA	6 (26)
10–15	NA	1 (14)	NA	6 (26)
Symptoms/signs before and at admission				
Temperature at admission, °C	7	38.0 (37.3–38.6)	22	38.0 (37.5–39.0)
Fever before admission	7	7 (100)	23	15 (65)
Headache	6	6 (100)	20	12 (60)
Seizures	7	2 (29)	23	14 (61)
Coma	3	1 (33)	17	10 (59)
Meningeal symptoms	7	7 (100)	23	19 (83)
Limb paralysis	7	1 (14)	21	4 (19)
Vomiting	6	6 (100)	18	11 (61)
Diarrhea	6	0 (0)	17	4 (24)
Days between disease onset and admission	7	2.0 (2.0–4.5)	21	3.0 (1.0–4.0)
Blood sample				
Leukocytes $\times 10^9$/L	7	16.3 (10.1–18.4)	22	17.1 (9.7–19.8)
Platelets/μL	7	226 (200–237)	19	263 (218–324)
Hemoglobin, g/L	6	62 (12–115)	11	14 (11–120)
Glucose, mmol/L	7	4.6 (3.6–6.1)	19	6.7 (4.2–8.5)
Glucose <3 mmol/L	7	1 (14)	19	1 (5)
CSF sample				
Leukocytes/mm^3	5	350 (200–350)	18	75 (16–225)
Lymphocytes/mm^3	4	45 (16–70)	11	40 (25–75)
Protein level >0.5 g/L	6	0 (0)	19	7 (37)
Transparent appearance of CSF	5	4 (80)	18	18 (100)
Liver enzymes at or after admission				
ALT, IU/L	7	31 (20–42)	14	21 (14–28)
AST, IU/L	7	43 (38–53)	14	38 (26–63)

*ALT, alanine aminotransferase; AST, aspartate aminotransferase; CSF, cerebrospinal fluid; IQR, interquartile range; NA, not applicable..

Technical Appendix Table 4. Correlation coefficients between initial variables and 3 principal components in study of hypoglycemic toxins and enteroviruses as causes of acute encephalitis-like syndrome in hospitalized children, Bac Giang Province, northern Vietnam, 2008–2011*

Variable	PC1	PC2	PC3
Age	−0.317	0.297	−0.768
Delay between disease onset and admission	0.290	0.803	0.015
Glucose level	0.528	0.130	0.409
Leukocytes in CSF	−0.096	0.803	0.118
ALT level	−0.794	−0.006	0.184
AST level	−0.771	0.175	0.398

*ALT, alanine aminotransferase; AST, aspartate aminotransferase; CSF, cerebrospinal PC, principal component.

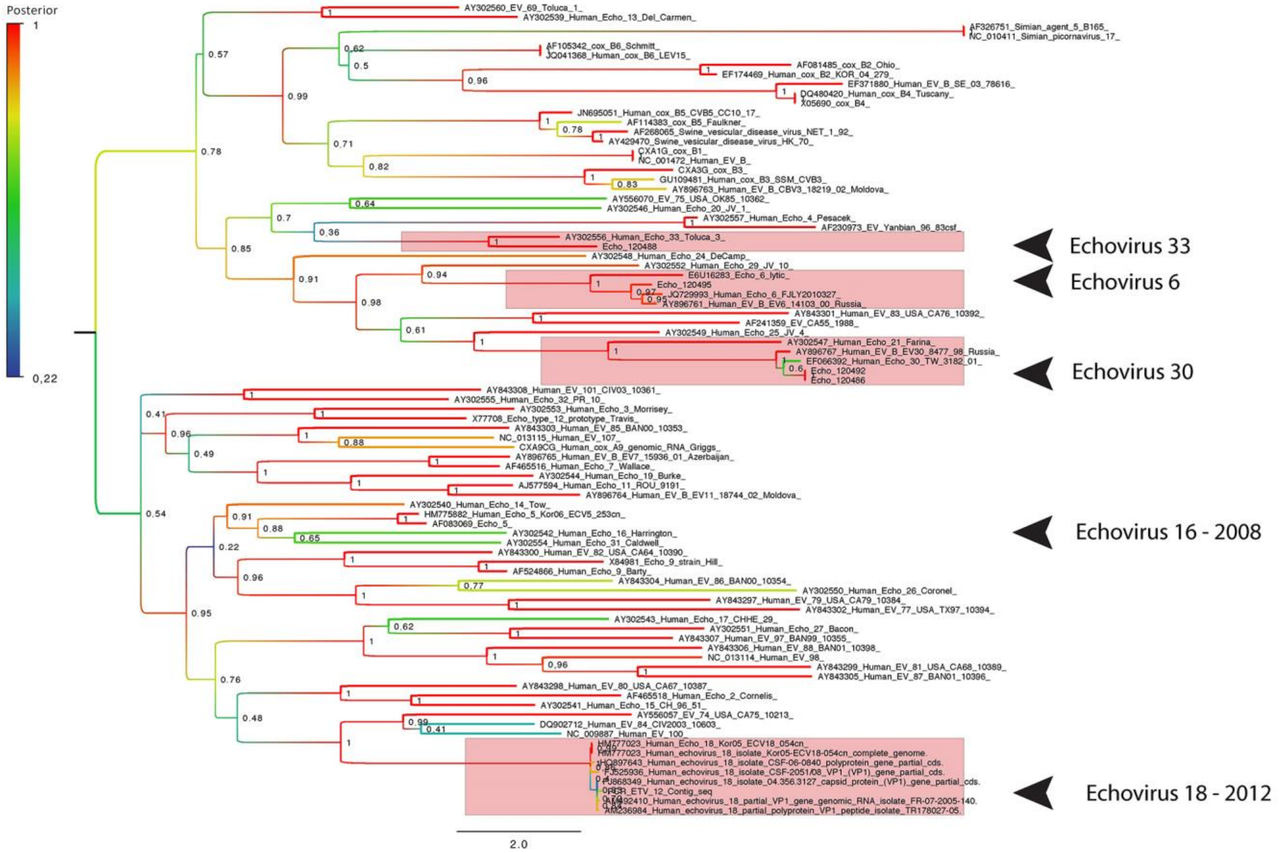

Technical Appendix Figure 1. Virus capsid protein 1–based phylogeny of enteroviruses, showing clustering of strains detected in this study with representatives of human enterovirus B serotypes. Numbers along branches are bootstrap values. Scale bar indicates nucleotide substitutions per site.

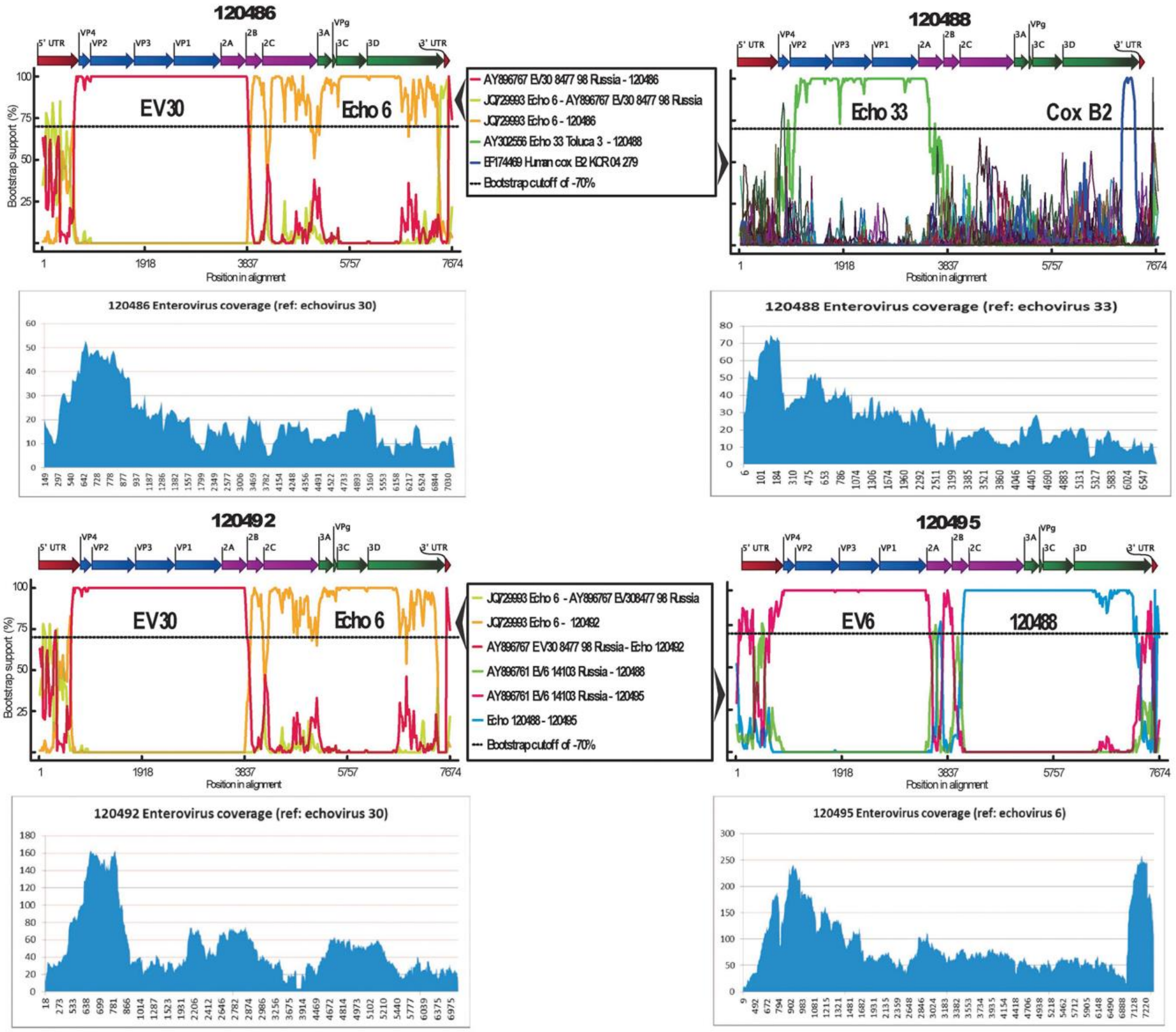

Technical Appendix Figure 2. Putative recombination patterns of enteroviruses 120486, 120488, 120492, and 120495 with other human enterovirus B strains and next-generation sequencing coverage of genomic sequences obtained from cerebrospinal fluid samples. For each enterovirus strain genomic sequence, the coverage is represented spanning genome, and the corresponding open reading frame map is depicted. Diagrams illustrate the association likelihood between sequences putatively involved in recombination events with the 4 genomes presented. Putative breakpoints were detected and parental sequences were identified on the basis of a combination of 5 algorithms (RDP, SiScan, BootScan, MaxChi, and Chimaera) applied to a matrix containing representative human enterovirus B genomes with a sliding window of 200 nt incremented every 20 nt. Echo, echovirus; EV, enterovirus; Cox, coxsackievirus; ref, referent; UTR, untranslated region. VP, virus capsid protein.

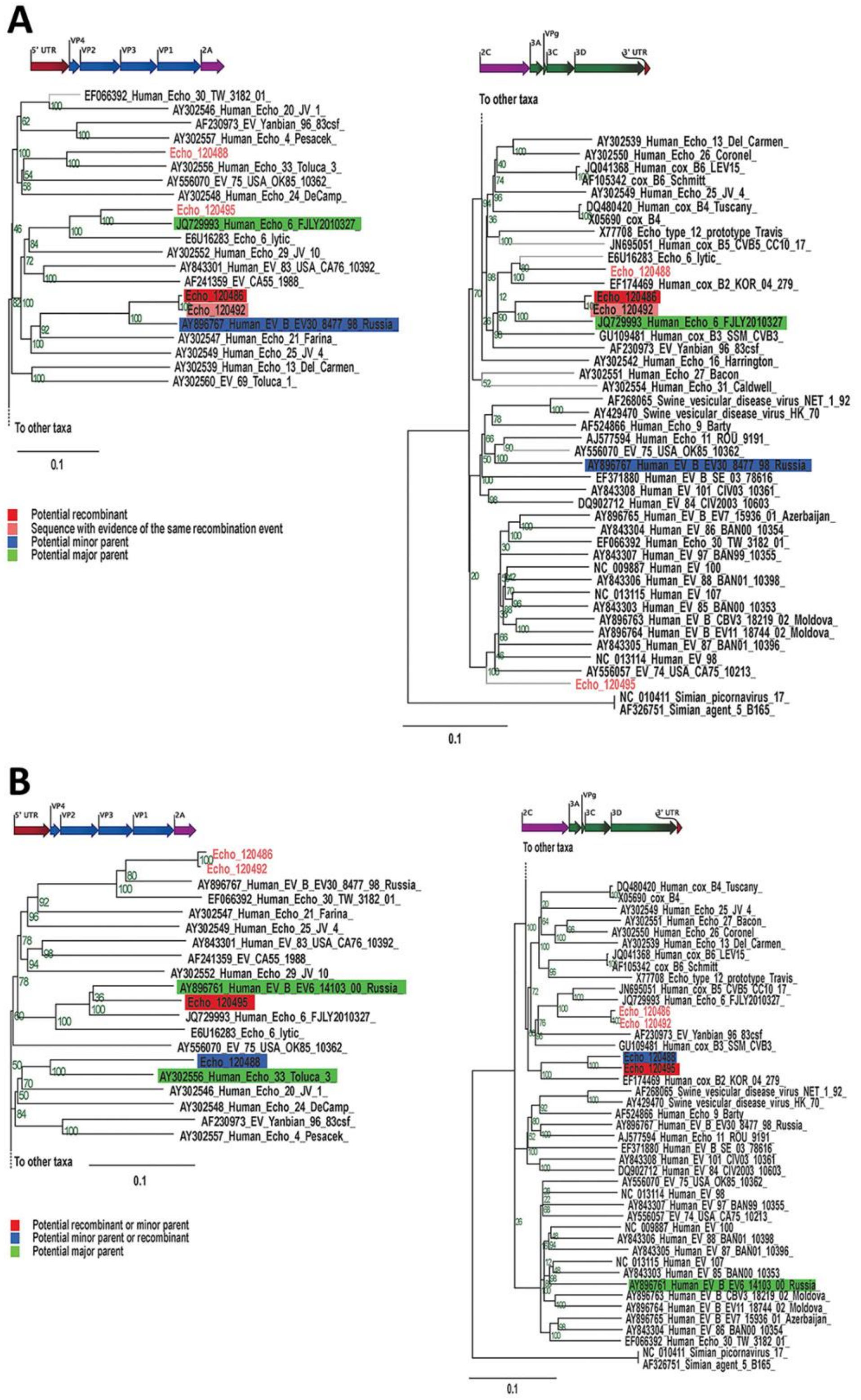

Technical Appendix Figure 3. Phylogenetic analysis of putative recombination patterns of enteroviruses detected. A) Phylogenies of beginning and end regions of genomes of human enterovirus B strains 12486 and 120492. B) Phylogenies of beginning and end regions of genomes of human enterovirus B strains 12488 and 120495. Clear incongruences emerge from phylogenetic reconstructions according to 3′ versus 5′ regions of the

genome, given the similarity of strains 120486 and 120492 and the proximity of strain 120486 with enterovirus 30 and the proximity of strain 120492 with echovirus 6 for VP (5′) and 2C to 3D (3′) genes. Similarities were observed between strains 120488 and 120495 in their 3′ genome regions, which contrasted with strong similarity of the VP (5′) gene of strain 120488 with enterovirus 33 and the VP (5′) gene of strain 120495 with enterovrius 6. Numbers along branches are bootstrap values. Scale bars indicate nucleotide substitutions per site. Cox, coxsackievirus; Echo, echovirus; EV, enterovirus; UTR, untranslated region. VP, virus capsid protein.

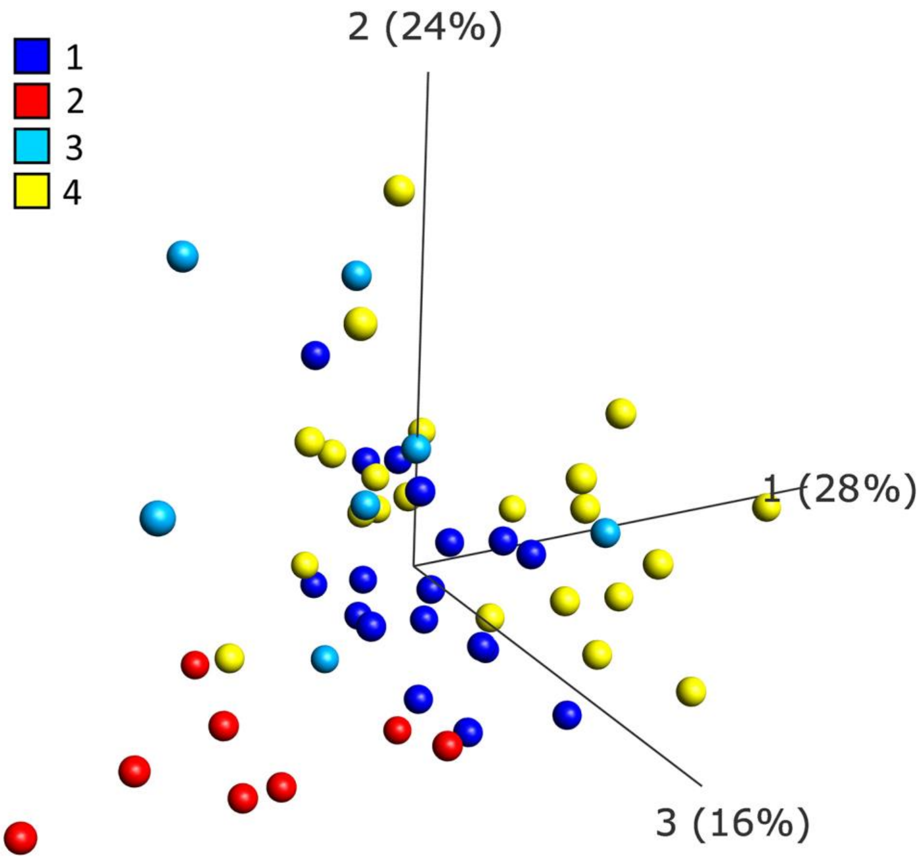

Technical Appendix Figure 4. Projection of patients in the 3-dimensional space formed by principal components. Colors indicate the 4 groups of patients described in our study. 1, patients positive for enteroviruses (n = 19) (dark blue); 2, patients positive for toxins (n = 8) (red); 3, patients negative for enterovirus and toxins (n = 7) (light blue); 4, patients negative for enterovirus and not tested for toxins (n = 23) (yellow).
